# Anesthesia-related unexpected cardiac arrest— What are we doing wrong on preoperative evaluation?

**DOI:** 10.1097/j.pbj.0000000000000157

**Published:** 2022-09-09

**Authors:** *Catarina Vieira, Filipa Sales, Inês Coles, Mariana M. Cunha

**Affiliations:** Department of Anaesthesiology, Unidade Local de Saúde de Matosinhos, Matosinhos, Portugal.

**Keywords:** anesthesia, cardiac arrest, cardiovascular risk stratification, functional capacity, heart failure

To the Editor,

Preoperative risk stratification is recommended as part of any strategy to prevent perioperative cardiovascular complications. Most algorithms proposed by several international guidelines emphasize the assessment of preoperative cardiopulmonary fitness, or functional capacity (FC), as an important component of estimating patients' risks for major cardiovascular morbidity and mortality. It is widely accepted that patients proceed directly to elective low and intermediate-risk noncardiac surgery if they are deemed capable of more than 4 metabolic equivalents (METs) of activity without symptoms, even in the presence of stable heart disease or clinical risk factors.^1^

Regarding a case of cardiac arrest after anesthesia induction, we propose a reflection on preoperative cardiac risk evaluation. Informed consent for publication was obtained.

A 63-year-old female, ASA III, with a history of obesity (BMI 31) and type 2 diabetes mellitus under insulin therapy and poor metabolic control, was scheduled for lumbar decompression surgery (intermediate-risk procedure). She attended a preanes-thetic evaluation 2 months prior to surgery when her FC was assessed. Her exercise capacity was difficult to estimate because of leg motor deficit and pain, but she denied angina pectoris, dyspnoea on exertion, orthopnea, or any other symptom suggestive of heart failure. Physical examination and preopera-tive testing found little to note and she had a previous normal resting transthoracic echocardiogram (Table 1). Cardiovascular stratification scores were also used: Revised Cardiac Risk Index revealed a 6% 30-day estimated risk of myocardial infarction, cardiac arrest, or death.

**Table 1:** Relevant test results: preoperative and postoperative

Blood analysis	Preoperative	Hb 13.0 g/dL | platelets 282 × 10^9^
		Cr 1.0mg/dL | K+ 5.0mmol/L | Na+ 136 mmol/L | HbA 1c 8.1%
		aPTT 29.3 s | PT 9.2s (INR 0.8)
	Postoperative	Hb 12.4g/dL | platelets 266 x 10^9^
		Cr 1.1 mg/dL| K+ 5.1 mmol/L | Na+ 133mmol/L | HbA 1c 10.8%
		High sensitivity Trop: 0h 334ng/L; 3h 280ng/L
		BNP 1780pg/mL | D-dimer 2368ng/mL
ECG	Preoperative	Regular sinus rhythm 82bpm, low voltage QRS
	Postoperative	Regular sinus rhythm 74bpm, poor R wave progression in V1–V3 but no ST-T acute changes
Transthoracic resting echocardiogram	Preoperative	Normal chamber sizes. Valvular structures with normal morphology. Preserved biventricular systolic function. LVEF of 55%
	Postoperative	Left ventricle with global hypokinesia and severe compromised function; bilateral pleural effusion

aPTT=activated partialthromboplastintime, BNP=brain natriuretic peptide, Cr=serum creatinine, ECG = electrocardiography, Hb=hemoglobin, HbA lc =glycated hemoglobin, INR= international normalized ratio, K+ = potassium, LVEF = left ventricular ejection fraction, Na+ = sodium, PT = prothrombin time, Trop=troponin.

General anesthesia was induced with a target-controlled infusion (TCI) of remifentanil (effect-site concentration 2ng/ mL) and propofol (total of 80 mg infused by TCI view mode),

followed by rocuronium for neuromuscular blockade. Tracheal intubation was easy. In a few minutes, it evolved to cardiorespi-ratory arrest in pulseless electrical activity, and reversion was achieved after 3 advanced life support cycles. Sedation was suspended and neuromuscular blockade reverted to allow neurological examination. She could follow commands and move both arms and legs. Arterial blood gas analysis revealed acidemia with hyperlactatemia and hypercapnia. Twelve-led electrocardiograms revealed poor R wave progression in V1–V3 but no ST-T acute changes. The patient was transferred to the intensive care unit (ICU) for stabilization and further investigation. Blood analysis showed no anemia or renal dysfunction and myocardial necrosis markers were just slightly elevated. Transthoracic echocardiogram revealed a left ventricle with global hypokinesia and severe compromised function and bilateral pleural effusion (Table 1). A thoracic computed tomography angiography excluded massive pulmonary embolism. Coronary angiography showed diffuse multivessel disease without culprit lesion, neither percutaneous nor surgical treatment was possible. The assumed diagnosis was cardiorespi-ratory arrest due to decompensated cardiac failure, previously unknown. Early extubation was possible and 2 days later she was transferred from the ICU. Hospital discharge occurred 11 days later, after compensation of the heart failure. Thirty days after the event she was alive and without further complications; medical follow-up to control cardiovascular risk factors was assured.

The selection of an appropriate preoperative test of FC should be individualized to the clinical scenario and surgery, as the identification of high- and low-risk patients allows planning of appropriate perioperative care. Although there is no clear agreement on the best single predictor of outcome, thresholds of peak oxygen consumption of 14mL/kg/min and oxygen consumption at the anerobic threshold of 11 mL/kg/min have been shown to select high-risk patients prior to noncardiac surgery.^2^

The usual standard of care for assessing preoperative FC involves a doctor making a subjective estimate after interviewing the patient. Although this approach is easily implementable into clinical practice, subjective assessment has limitations, including little agreement with validated measures of FC and poor accuracy when predicting both death or complications after noncardiac surgery.^3^

A significant number of studies raise concerns about using this method to evaluate preoperative exercise capacity, such as the METs study.^4^ The investigators compared subjective assessment of FC with alternative objective markers, such as cardiopulmo-nary exercise testing (CPET) and scores on Duke Activity Status Index (DASI) questionnaire, for predicting death or complications after elective non-cardiac surgery. DASI is a simple self-administered questionnaire correlated with gold-standard measures of FC. Their results showed that subjective assessment consistently performed poorly, revealing low sensitivity for identifying peak oxygen consumption of less than 14 mL/kg/min and no association with postoperative outcomes.^4^ CPET did not improve most aspects of preoperative risk assessment.^4^ On the other hand, DASI scores were positively correlated with peak oxygen consumption and were also the only marker associated with predicting the primary outcome of myocardial infarction or death.^4^ Also, the MET-REPAIR, an international multi-center study, is being developed by the European Society of Anaesthesi-ology to investigate this problem.

In this case, the preanesthetic evaluation was misleading, because, despite the patient's poor FC, she denied symptoms suggestive of ischemic heart disease, having only diabetes as a clinical risk factor. For this, we believe that in patients with uncertain FC due to preexisting conditions, an evaluation as objectively as possible is of utmost importance, and we question ourselves about the validity of most practice guidelines when it comes to these patients. The simple DASI questionnaire can be easily implemented into most perioperative practice settings, and it would be an important tool in improving preoperative evaluation, as it also measures different components of low FC, such as musculoskeletal conditions and frailty.^5^ It is also a useful pretest tool to determine a patient’s ability to achieve appropriate METs and has the potential to guide the selection of stress cardiac testing if necessary.^6^

In conclusion, this report alerts for the inaccuracy of subjective preoperative assessment of FC. It should not be used in clinical practice because it does not accurately identify patients with poor fitness and those at increased risk for morbidity and mortality after surgery. More objective measures, such as DASI questionnaires and CPET, may be considered in the assessment of perioperative cardiac risk. We believe that new measures to access cardiovascular fitness are needed, new guidelines should address the difficulty to identify some high-risk patients and propose orientation to manage these situations.
